# Mid-lumbar traumatic spondyloptosis without neurological deficit: A case report and literature review

**DOI:** 10.1097/MD.0000000000019578

**Published:** 2020-03-20

**Authors:** Feng Xu, Zhisen Tian, Changfeng Fu, Liyu Yao, Mengjie Yan, Congcong Zou, Yi Liu, Yuanyi Wang

**Affiliations:** a Department of Spine Surgery, The First Hospital of Jilin University; b Department of Orthopedics, China-Japan Union Hospital Affiliated to Jilin University; c Department of Pediatric Surgery, The First Hospital of Jilin University; d Department of Internal Medicine Cardiology, China-Japan Union Hospital Affiliated to Jilin University, Changchun, Jilin, China.

**Keywords:** fracture dislocation, mid-lumbar trauma, saving fracture, spondyloptosis

## Abstract

**Introduction:**

Spondyloptosis is a form of vertebral dislocation and the most advanced form of spondylolisthesis. Traumatic spondyloptosis is usually caused by high-energy impact and results in unstable spine deformity and spinal canal deformation, which lead to severe spinal cord injury. Traumatic spondyloptosis is mostly reported in the lumbo-sacral junction, while it is rarely documented in mid-lumbar segments. To the best of the authors’ knowledge, only 16 cases of mid-lumbar spondyloptosis have been described previously. Herein, we present a L3 to L4 spondyloptosis case that did not involve neurological deficit.

**Patient concerns:**

A 42-year-old man presented to the emergency department after an accident involving a fall. The patient developed severe back pain and spinal deformity, while his neurologic function remained intact. Radiological examinations indicated complete posterior vertebral dislocation at L3 to L4 and a fracture at the bilateral pelvic ischial tuberosity without major vessel injury or severe dura sac compression.

**Diagnoses:**

L3 to L4 complete vertebral dislocation, pelvic ischial tuberosity fracture.

**Interventions:**

For treatment, the patient underwent fracture reduction, L3 to L4 intervertebral fusion, and internal fixation 7 days post-injury.

**Outcomes:**

Postoperative digital radiography showed the correction of the spinal deformity. The patient was pain-free and fully rehabilitated 3 months after the surgery. At the 1-year follow-up, the patient was completely asymptomatic and had achieved normal alignment.

**Conclusions:**

We reported an L3 to L4 traumatic spondyloptosis case that involved intact neurology, which is the first-ever reported mid-lumbar spondyloptosis case that involved complete posterior column and neural sparing. For the treatment of traumatic spondyloptosis without neurological deficit, restoring stability and preventing secondary cord injury should be taken into consideration.

## 1 Introduction

Traumatic spondyloptosis is defined as >100% traumatic subluxation of one vertebral body in the sagittal or coronal plane.^[1]^ Implicated by high-energy impact, spondyloptosis results in unstable spine deformity and spinal canal deformation, which often lead to para-lesion damage and spinal cord injury. In the lower back, traumatic spondyloptosis frequently occurs at the thoraco-lumbar or lumbo-sacral junctions, while it has rarely been reported at the mid-lumbar level.^[2]^ Herein, we present an L3 to L4 spondyloptosis case that did not involve neurological deficit, which is the first neurologically intact mid-lumbar spondyloptosis case reported, and discuss the injury mechanism and applied treatment.

## 2 Case report

A 42-year-old mentally handicapped man presented to the emergency department after an indescribable accident involving a fall while working in a welfare factory without a witness. The patient developed severe back pain and spinal deformity, but maintained normal neurological functions in his lower extremities. No significant sensory abnormality was noted. He could even sit up from his bed when his mental state was unstable.

**Figure 1:**
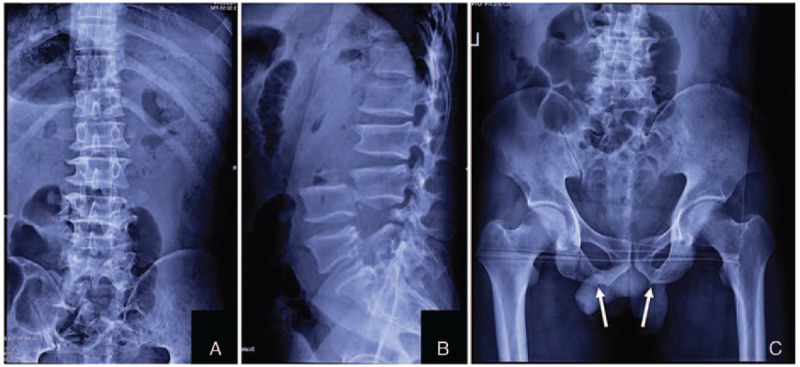
Preoperative anteroposterior (A) and lateral (B) radiograph show complete posterior vertebral dislocation (spondyloptosis) at L3 to L4 and pedicle fractures at L4 to S1. Preoperative anteroposterior radiograph shows the fracture at bilateral pelvic ischial tuberosity (arrow).

**Figure 2:**
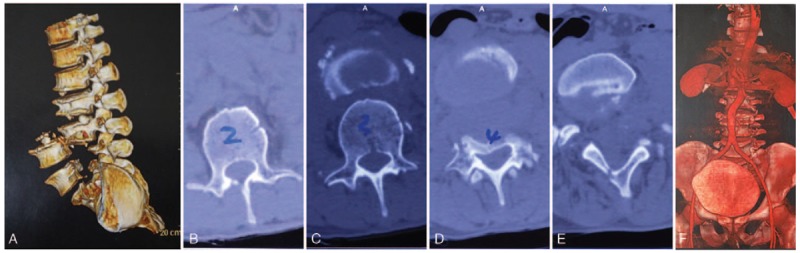
Three-dimensional reconstruction of demonstrates pedicle disruptions at L4 to S1 with intact neural arches (A). Axial computed tomography shows the space of spinal canal was maintained at injured segments (B–E). Abdominal angiography reveals the major vessels were not injured.

**Figure 3:**
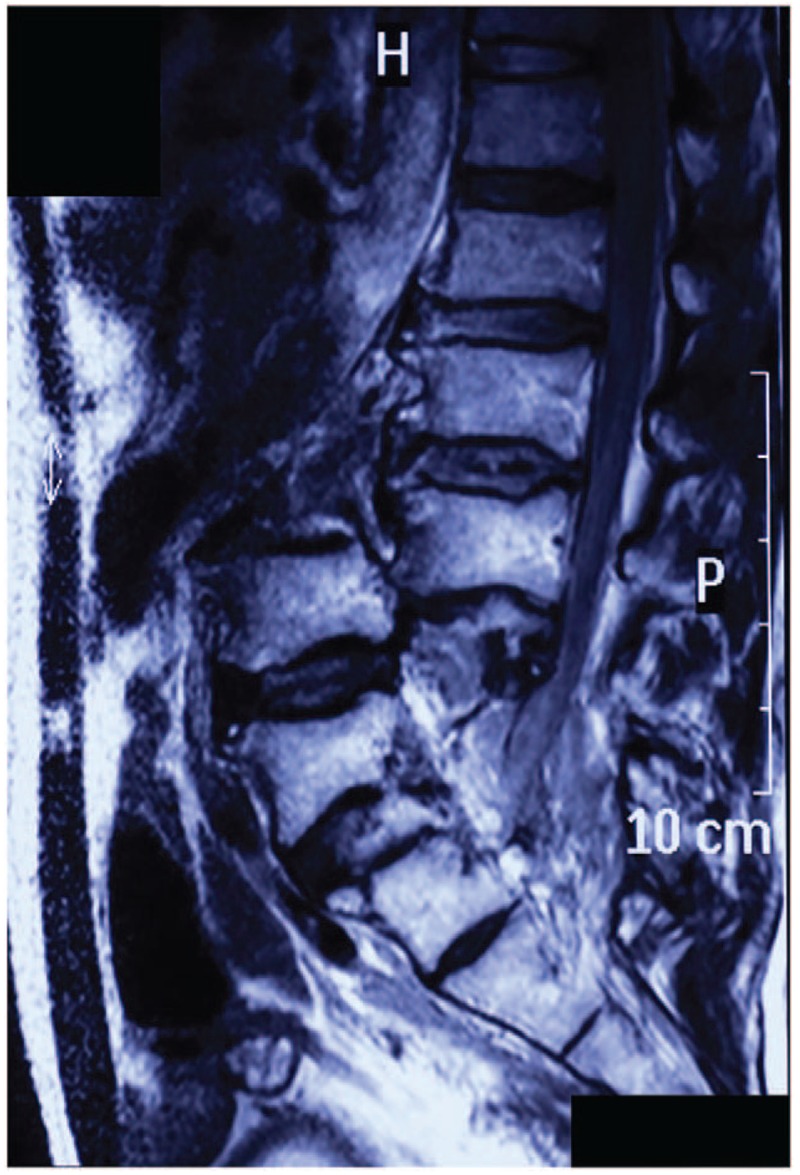
T2-weighted sagittal magnetic resonance imaging shows the neural arches at injured segments are barely damaged and the dura sac at corresponding segments is mildly compressed.

Digital radiography (DR) showed pedicle fractures at L4 to S1, complete posterior vertebral dislocation at L3 to L4, and fractures at the bilateral pelvic ischial tuberosity (Fig. 1). Computed tomography showed pedicle disruptions with an intact neural arch, which maintained the space of the spinal canal at the injured segments (Fig. 2A–E). Abdominal angiography excluded major vessel injury (Fig. 2F). Magnetic resonance imaging indicated only mild dura sac compression at the corresponding segments, and the integrity of the neural arch was not damaged (Fig. 3).

For treatment, the patient underwent fracture reduction, L3 to L4 intervertebral fusion, spinal canal exploration and internal fixation 7 days post-injury. L2, L3, and the right pedicle screw of S1 were implanted per routine, while L5 internal fixation was excluded because of the severe pedicle fracture, and 2 sacroiliac screws were adopted instead of the left pedicle screw of S1 for the same reason. In L4, bilateral pedicle screws were implanted in the pedicle stump of L4 vertebral body. Manual reduction was performed by lifting the screw crown, and connecting rods were applied. Afterward, allogeneic bone was grafted for L3 to L4 intervertebral fusion. During the procedure, the patient's posterior elements were furthest preserved, except for part of the L3 inferior laminae and L4 superior laminae that were removed and bilateral facets that were resected for L4 fixation and spinal canal exploration.

**Figure 4:**
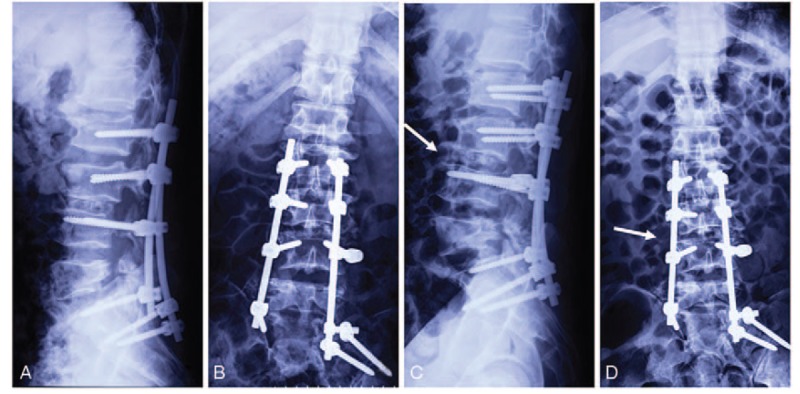
Postoperative anteroposterior (A) and lateral (B) digital radiographs show the spinal deformity was corrected. The digital radiographs at 1-year follow-up (C and D) show the posterior reduction with instrumentation achieved normal alignment, and the intravertebral fusion was effective (arrow).

The postoperative course was favorable. Postoperative DR showed correction of the spinal deformity (Fig. 4A and B). The patient was pain-free and fully rehabilitated 3 months after the surgery. At the 1-year follow-up, the patient was completely asymptomatic and had achieved normal alignment (Fig. 4C and D).

## 3 Discussion

Spondyloptosis is a form of vertebral dislocation and the most advanced form of spondylolisthesis. Under high-energy impact, one segment is lodged in the axial space of the adjacent vertebral body.^[1]^ Mid-lumbar spondyloptosis is an extremely exceptional injury caused by high-energy trauma, mostly associated with traffic accidents and falls.^[2]^ Because of the anatomical structure of spine, the thoraco-lumbar junction (T12–L2) is more frequently implicated by the lesion of fracture dislocation, while spondyloptosis is more often reported in the lumbo-sacral junction.^[3]^ In mid-lumbar segments (L2–L4), vertebral dislocation is rarely documented; with the current case, only 16 cases have been reported since 1966 (Table 1).

**Table 1:**
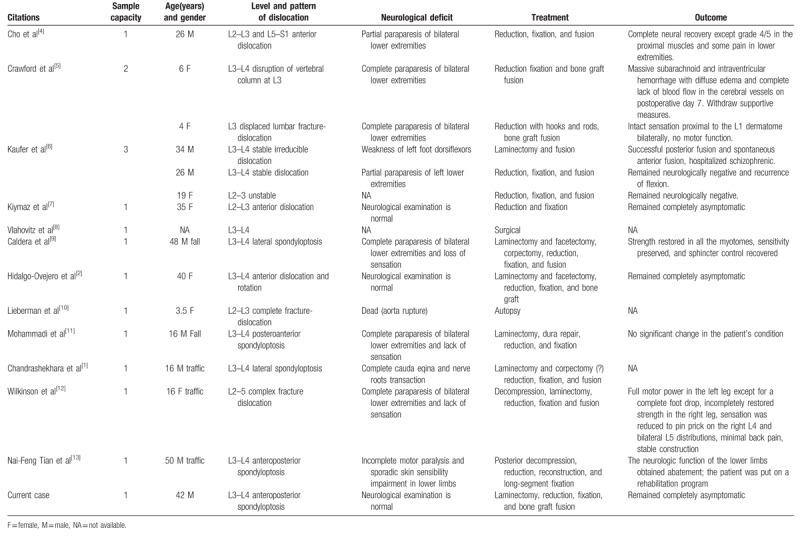
Reported fracture dislocation in mid-lumbar segments.

**Figure 5:**
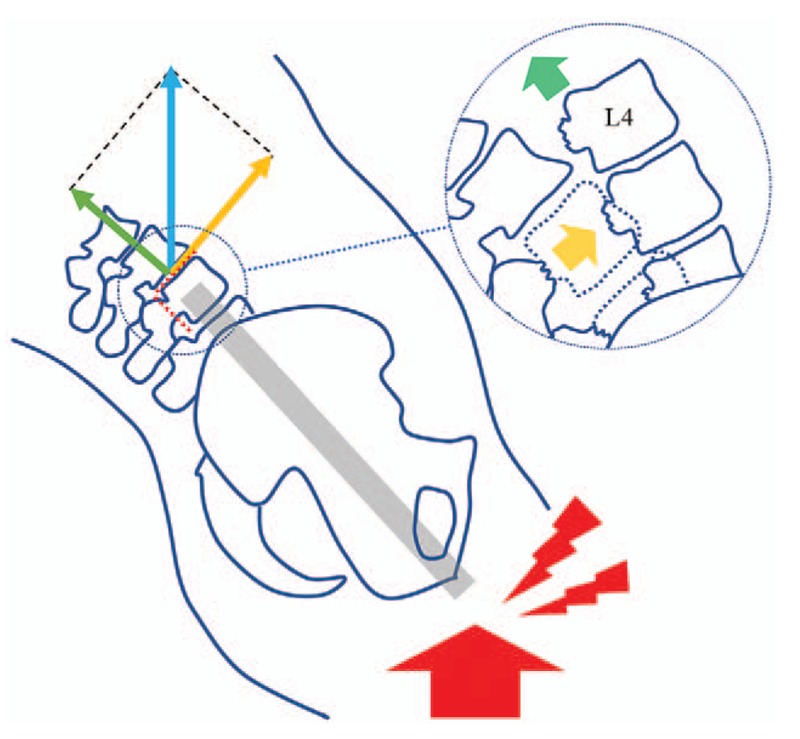
The sketch of the injury mechanism in this case. The red arrow shows the vertical impact force. The blue arrow shows the impact force at L3 to L4 level conducted through vertebral bodies. The yellow arrow and the green arrow show the components of impact force at L3 to L4 level in different directions.

Among these cases, 5 patients conformed to more than 100% subluxation and were diagnosed with traumatic spondyloptosis. The rarity of mid-lumbar spondyloptosis may be attributed to the relatively rigid anatomic structure and high immediate mortality caused by combined trauma, such as aortic injury and cerebral trauma.^[10]^ In the present case, with clues provided by his co-workers and the situation of his lumbar and ischium injury, we speculated that the falling injury occurred when the patient leaned against an elevator that suddenly started moving, and the fracture was induced by extension and shearing violence (Fig. 5).

According to the Denis spine fracture classification, fracture dislocation has 3 types: flexion dislocation, flexion–rotation, and shear.^[14]^ Dislocation of a vertebral body is unlikely to be induced by hyperflexion or hyperextension alone, but by the combined effect of shearing and rotational force with sagittal hypermobility.^[15]^ Denis further divided the shearing type into the posteroanterior and anteroposterior subtypes,^[16]^ and the current case conforms to the latter subtype, which is usually caused by hyperextension and shearing force and results in fractures in the posterior complex and pedicles.^[17]^ By analyzing the trend in spinal fractures, we hypothesized the injury mechanism as follows (Fig. 5). The patient fell from a height with a flexed hip joint and extended lumbar spine. With this unique position, the L3 to L4 disc was at a certain angle to the ground. The axial impact force (red arrow) was mainly conducted through the vertebral body (blue arrow). At the L3 to L4 level, the component that was parallel to the disc (yellow arrow) caused transverse damage to the disc and pedicle fracture, which resulted with the split between posterior elements and vertebral bodies. The component of the impact force that was perpendicular to the L3 to L4 disc (green arrow) pushed the dislocated vertebral bodies to the cranial side and lodged L4 anterior to L3.

As a severe spinal fracture, spondyloptosis is usually combined with spinal cord injury, the severity of which differs in separated segments. Mishra et al reported that as many as 80% of patients with spondyloptosis develop complete paraplegia, and very few well-documented cases involve neurologically intact patients.^[16]^ Among the reported mid-lumbar dislocation cases, 6 cases involved complete paraplegia, 4 cases involved varying degrees of partial paraparesis, and 3 cases including ours did not involve neurological deficits (Table 1). In the present case, fracture of the bilateral pedicle separated the vertebral body and posterior elements and enlarged the spinal canal, and the free-floating neural arch further preserved the dura sac. The majority of cases that do not involve neurological deficit have similar saving fractures in the unilateral or bilateral pedicles, facets, and lamina.^[18,19]^ This spontaneous decompression mechanism is considered the most important factor that leads to spared neural function in fracture dislocation cases.^[20]^ Interestingly, Rahimizadeh et al reported normal neurologic function in a case of complete fracture dislocation without a saving fracture, in which the spinal cord acted like a hinge of the rotated vertebral body and remained uninjured.^[17]^

Fracture dislocation is the most common unstable spinal injury that usually involves three columns.^[11]^ Nonetheless, dislocation with a saving fracture sometimes results in a less injured neural arch. In this case, the shearing force was neutralized by the pedicle fracture and dislocation of vertebral bodies, which preserved the posterior column element. As a result, the patient was able to sit up when mental instability occurred. The intact posterior column could be supportive, which demonstrated the posterior column's important role in spinal stability. Unlike the traditional perspective that considers the posterior elements as a “tension band,” as well as the other columns that lift the weight,^[21,22]^ the present case indicated that the posterior elements contribute to spinal stability in all directions. However, in spinal surgery, in the current case as well, the integrity of the posterior elements is always sacrificed for the decompression of the spinal canal or reconstruction of axial stability of the anterior column.

For spondyloptosis, surgical treatment is essential for re-establishing spinal alignment, restoring stability, and spinal canal decompression; however, the treatment of fracture dislocation without neurological deficit remains debatable. In the case series reported by Mishra et al and Chandrashekhara et al, a total of 23 of 24 cohorts underwent surgery via a posterior approach with fixation, fusion, and reduction with/without laminectomy or corpectomy. Despite most patients achieving complete reduction, the neurological outcomes were unfavorable for those with devastating primary cord injury.^[1,16]^ Gitelman et al reported a decompression procedure in a neurologically intact thoracic spondyloptosis case in which they performed posterior laminectomy and in situ fusion on adjacent vertebra without reduction attempts.^[23]^ Although there was no neurological deficit, Gitelman et al insisted laminectomy for exploration of hematoma and latent compression. The main disadvantage of this surgical strategy is the introduction of posterior instability with an unfixed anterior column.^[23]^ Yamaki et al used an anterior–posterior approach in a pediatric patient with lumbo-sacral spondyloptosis with slight foot weakness. In the procedure, the patient first underwent anterior manual reduction and fixation, and laminectomy and adjacent pedicle screw fixation were then conducted via a posterior approach, which provided adequate realignment and expansion of the spinal canal.^[24]^ Conservative treatment was also reported by clinicians in adolescent patients with spondyloptosis without neurological deficit; despite the outcomes involved being pain-free and neurologically intact, the patient developed residual spine deformity and long-term back pain.^[25]^

In this case, we conducted posterior decompression and reduction with fixation of the adjacent and 1 injured vertebral bodies. Because of the extra damage induced to the complete posterior column, laminectomy and facetectomy might be a controversial part of the surgical strategy. We had 3 reasons for the procedure:

1. laminectomy prevents latent compression, including hematoma and bony fragments;
2. with laminectomy and facetectomy, manual reduction can be performed under direct vision, and it also avoids new compression induced by reduction; and
3. without the blocking floating laminar, internal fixation could be implemented for the injured vertebral bodies, which aided reduction and rebuilding of alignment and stability.

## 4 Conclusions

In summary, traumatic spondyloptosis is a rare fracture related to high-energy impact and usually leads to devastating clinical consequences. Herein, we present an L3 to L4 traumatic spondyloptosis case that did not involve neurological deficit, which is the first-ever reported mid-lumbar spondyloptosis case that involved complete posterior column and neural sparing. For treatment, restoring stability and preventing secondary cord injury might be the principle of traumatic spondyloptosis without neurological deficit, which achieved favorable outcomes in our case.

## Author contributions

**Conceptualization:** Yi Liu, Yuanyi Wang.

**Data curation:** Mengjie Yan, Congcong Zou.

**Funding acquisition:** Yuanyi Wang.

**Investigation:** Zhisen Tian, Changfeng Fu, Yuanyi Wang.

**Methodology:** Feng Xu, Changfeng Fu, Yuanyi Wang.

**Resources:** Liyu Yao, Yuanyi Wang, Xu Feng.

**Supervision:** Yi Liu, Yuanyi Wang.

**Visualization:** Liyu Yao.

**Writing – original draft:** Zhisen Tian, Yuanyi Wang.

**Writing – review & editing:** Yi Liu, Yuanyi Wang.

Yuanyi Wang orcid: 0000-0002-9191-3782.

## References

[1] Chandrashekhara SH Kumar A Gamanagatti S Unusual traumatic spondyloptosis causing complete transection of spinal cord. Int Orthop 2011;35:1671–5. 21221578 10.1007/s00264-010-1190-6 PMC3193952

[2] Hidalgo-Ovejero AM Garcia-Mata S Martinez-Lecea FJ L3-L4 dislocation without neurological lesions. Bull NYU Hosp Joint Dis 2010;68:60–4. 20345367

[3] Yadla S Lebude B Tender GC Traumatic spondyloptosis of the thoracolumbar spine. J Neurosurg Spine 2008;9:145–51. 18764746 10.3171/SPI/2008/9/8/145

[4] Cho SK Lenke LG Hanson D Traumatic noncontiguous double fracture-dislocation of the lumbosacral spine. Spine J 2006;6:534–8. 16934723 10.1016/j.spinee.2006.01.015

[5] Crawford CH 3rd Puno RM Campbell MJ Surgical management of severely displaced pediatric seat-belt fracture-dislocations of the lumbar spine associated with occlusion of the abdominal aorta and avulsion of the cauda equina: a report of two cases. Spine 2008;33:E325–8. 18449033 10.1097/BRS.0b013e31816f6c56

[6] Kaufer H Hayes JT Lumbar fracture-dislocation. A study of twenty-one cases. J Bone Joint Surg Am 1966;48:712–30. 15580739

[7] Kiymaz N Yilmaz N Mumcu C Traumatic lumbar fracture-dislocation related to spina bifida occulta: case report. Hiroshima J Med Sci 2005;54:57–9. 15991599

[8] Vlahovitch B Fuentes JM Choucair Y Fractures and dislocations of the lumbar vertebrae (author's transl). J Chir 1978;115:659–62. 744772

[9] Caldera G Cahueque M Recovery after fracture dislocation of L3/L4 ASIA B: case report. Trauma Case Rep 2016;2:9–15. 29942833 10.1016/j.tcr.2016.03.003 PMC6011859

[10] Lieberman I Chiasson D Podichetty VK Aortic disruption associated with L2-L3 fracture-dislocation in a case of child abuse: a case report. J Bone Joint Surg Am 2010;92:1670–4. 20595576 10.2106/JBJS.I.01404

[11] Mohammadi HR Zandi S Complete traumatic fracture-dislocation L3-L4 of the lumbar spine. Pakistan J Med Sci 2013;29:1283–4. 10.12669/pjms.295.3783 PMC3858937 24353738

[12] Wilkinson JS Riesberry MA Mann SA Traumatic lateral expulsion of the L-4 vertebral body from the spinal column. J Neurosurg Spine 2011;14:508–12. 21275552 10.3171/2010.11.SPINE091028

[13] Tian NF Mao FM Xu HZ Traumatic fracture-dislocation of the lumbar spine. Surgery 2013;153:739–40. 22575882 10.1016/j.surg.2012.03.008

[14] Denis F The three column spine and its significance in the classification of acute thoracolumbar spinal injuries. Spine 1983;8:817–31. 6670016 10.1097/00007632-198311000-00003

[15] Robert R A study of the mechanics of spinal injuries. J Bone Joint Surg Brit 1960;42-B:810–23.

[16] Mishra A Agrawal D Gupta D Traumatic spondyloptosis: a series of 20 patients. J Neurosurg Spine 2015;22:647–52. 25768668 10.3171/2014.10.SPINE1440

[17] Rahimizadeh A Asgari N Rahimizadeh A Complete thoracolumbar fracture-dislocation with intact neurologic function: explanation of a novel cord saving mechanism. J Spinal Cord Med 2018;41:367–76. 28648115 10.1080/10790268.2017.1336300 PMC6055955

[18] Shapiro S Abel T Rodgers RB Traumatic thoracic spinal fracture dislocation with minimal or no cord injury. Report of four cases and review of the literature. J Neurosurg 2002;96: 3 Suppl: 333–7. 11990843 10.3171/spi.2002.96.3.0333

[19] Enishi T Katoh S Sogo T Surgical treatment for significant fracture-dislocation of the thoracic or lumbar spine without neurologic deficit: a case series. J Orthop Case Rep 2014;4:43–5. 27298981 10.13107/jocr.2250-0685.194 PMC4719325

[20] Hsieh CT Chen GJ Wu CC Complete fracture-dislocation of the thoracolumbar spine without paraplegia. Am J Emerg Med 2008;26:633.e5–7. 10.1016/j.ajem.2007.09.023 18534310

[21] Asmussen E Klausen K Form and function of the erect human spine. Clin Orthop 1962;25:55–63. 13965250

[22] Whitesides TE Jr Traumatic kyphosis of the thoracolumbar spine. Clin Orthop Relat Res 1977;78–92. PMID: 340100. 340100

[23] Gitelman A Most MJ Stephen M Traumatic thoracic spondyloptosis without neurologic deficit, and treatment with in situ fusion. Am J Orthop (Belle Mead, NJ) 2009;38:E162–5. 20011746

[24] Yamaki VN Morais BA Brock RS Traumatic lumbosacral spondyloptosis in a pediatric patient: case report and literature review. Pediatr Neurosurg 2018;53:263–9. 29847821 10.1159/000488766

[25] Goni V Gopinathan NR Saini UC Traumatic L5 over S1 spondyloptosis without neurological involvement managed nonoperatively: a case report. Chin J Traumatol 2013;16:178–81. 23735554

